# Clinical, endoscopic, pathological characteristics and management of cap polyposis: experience from a Tertiary Hospital in China

**DOI:** 10.3389/fphar.2024.1391367

**Published:** 2024-05-09

**Authors:** Yi Lu, Lingyu Huang, Xiaoying Lou, Chunyu Chen, Jiachen Sun

**Affiliations:** ^1^ Department of Gastrointestinal Endoscopy, The Sixth Affiliated Hospital, Sun Yat-sen University, Guangzhou, China; ^2^ Guangdong Provincial Key Laboratory of Colorectal and Pelvic Floor Diseases, The Sixth Affiliated Hospital, Sun Yat-sen University, Guangzhou, China; ^3^ Biomedical Innovation Center, The Sixth Affiliated Hospital, Sun Yat-sen University, Guangzhou, China; ^4^ Department of Pathology, The Sixth Affiliated Hospital, Sun Yat-sen University, Guangzhou, China; ^5^ Department of Gastrointestinal Surgery, The Sixth Affiliated Hospital, Sun Yat-sen University, Guangzhou, China

**Keywords:** cap polyposis, *Helicobacter pylori*, treatment, colonoscopy, resection

## Abstract

**Background and aims:**

Cap polyposis (CP) is a rare kind of benign disease, and the majority of previously published relevant articles involve a small number of patients. Hence, we summarized our experience to contribute additional data, hoping to raise awareness of this disease.

**Methods:**

From 1 January 2017 to 1 November 2021, consecutive patients diagnosed with CP were retrospectively reviewed. Their medical histories, and laboratory, imaging, endoscopic, and pathology results were analyzed. We made telephone calls to the patients and searched for the information in our electronic medical records to obtain the follow-up results.

**Results:**

Forty-one patients were chosen for analysis. The median age of the patients was 20 years old, and 90.24% (37 patients) of the patients were male. The majority of the patients presented with hematochezia. The rectum was the most commonly affected site, and the *Helicobacter pylori* infection rate was high. There were multiple and combined treatments for these patients. These treatments can be divided into 3 main categories: medical therapy, endotherapy and surgery. Medical therapy helped to diminish the size of but the polyps were difficult to resolve; however, the patients’ symptoms could be diminished. Twenty-three patients underwent surgical resection, and 12 patients received endotherapy. We further compared the two methods of polyp resection. Both endotherapy and surgery were safe, and the recurrence risk was not significantly different between the two kinds of therapy (*p* = 0.321).

**Conclusion:**

The clinical improvement of medical treatments was not satisfactory, and endotherapy or surgical resection could remove the polyposis and provide temporary relief, but the recurrence rates were high.

## Introduction

Cap polyposis (CP) is a rare benign disease that usually occurs in the rectum and sometimes in the colon or stomach (GT et al., 1985; Esaki et al., 2001; Oiya et al., 2002; Da et al., 2011; Brunner et al., 2019). CP was first described by Williams et al., in 1985, and as the name implies, polyps have a “cap” formed by granulation tissue covered with fibrinopurulent exudates (GT et al., 1985). The etiology of CP is still unclear and might be associated with mucosal prolapse, inflammation, infection with *H. pylori* (*Helicobacter pylori*) or other microorganisms, and dysbacteriosis (Konishi et al., 2005; Suzuki et al., 2014; Murata et al., 2017; Okamoto et al., 2018; Brunner et al., 2019). The clinical manifestations of CP are constipation, diarrhea, abdominal pain, mucous stools, hematochezia, and hypoproteinemia (Campbell et al., 1993; Oshitani et al., 1995; Brunner et al., 2019). Endoscopically, it is common to find multiple erythematous, sessile polyps covered by fibrinopurulent exudates of different sizes, and they are often located at the apices of the transverse mucosal folds with normal mucosa between the polyps. These characteristics help to differentiate CP from inflammatory bowel disease (IBD) and cancer via endoscopy (Aggarwal et al., 2021). On imaging, CP is sometimes misdiagnosed as cancer, and surgery might be subsequently suggested to the patient. Hence, improving the diagnostic accuracy before surgery is very important. There are a variety of therapies for CP, such as *H. pylori* eradication, antibiotics, steroids, 5-amino salicylic acids (5-ASAs), infliximab, and endoscopic or surgical resection (Maunoury et al., 2005; Suzuki et al., 2014; Brunner et al., 2019). However, to date, no standard therapy has been recommended for CP, as sometimes the polyps are not resolved or reoccur (Brunner et al., 2019). The majority of previously published articles on this issue are case reports or case series with a small number of patients; hence, we summarized the experience in our hospital to contribute additional data, hoping to increase the physicians’ and surgeons’ awareness of this disease.

## Methods

### Patients

The data of consecutive patients who were diagnosed with CP in our hospital between 1 January 2017 to 1 November 2021, were retrospectively reviewed. The patients were identified from the pathology database in our hospital. The inclusion criteria were as follows: a. patients with confirmed CP, in which endoscopy revealed multiple polyps covered by fibrinopurulent exudates, and pathology revealed inflammatory polyps covered by a thick layer of fibrinopurulent exudate, forming the “cap” structure; and b. patients whose clinical information could be obtained. The exclusion criteria were as follows: a. foreigners; b. patients without endoscopic images; c. patients who refused to receive any treatment in our hospital; d. patients with IBD; and e. patients with parastomal polyps. The study protocol was approved by the Institutional Review Board (IRB) of The Sixth Affiliated Hospital, Sun Yat-sen University (2021ZSLYEC-455), and the requirement for signed informed consent was waived by the IRB.

### Information collected

The following information was collected from our electronic medical records: age, sex, symptoms, medical history, laboratory test results [mainly hemoglobin, platelet, white blood cell, neutrophil, serum albumin (ALB), and high-sensitivity C reactive protein (hs-CRP)], imaging results [contrast-enhanced abdominal computed tomography (CT) or magnetic resonance imaging (MRI)], endoscopic findings (mainly gastroduodenoscopy and ileocolonoscopy), rectal ultrasound, pathology results, final diagnosis, and treatment results. We made telephone calls to the patients and searched for information in our electronic medical records to obtain the follow-up results of the patients.

### Statistical analysis

IBM SPSS Statistics Version 22 was used to perform the statistical analyses. Continuous variables with normal distribution are presented as mean (standard deviation, SD), and those without normal distribution are presented as median (interquartile range, IQR). Categorical variables are expressed as numbers (percentages), and they were tested by using the χ^2^ test. To compare the recurrence rates and durations of endotherapy and surgical resection, we used the Kaplan‒Meier method to analyze the recurrence rates. The endpoint was defined as the first recurrence since endotherapy/surgical resection, and recurrence was defined as polyps that recurred on endoscopy or on digital rectal examination, or if symptoms reappeared (as not all patients underwent endoscopic reexamination).

## Results

### Demographic and clinical characteristics

We selected 41 patients with CP for final analysis, among whom, 7 patients had previously received endotherapy or surgery at other hospitals. The basic information, laboratory results, and imaging characteristics of the patients are presented in Table 1.

**TABLE 1:** Basic information, laboratory and imaging characteristics.

	Results
Sex, M (n, %)	37 (90.24%)
Age, years (median, IQR)	20 (14)
Symptoms (n, %)^a^	
Hematochezia	29 (70.73%)
Increased stool frequency	5 (12.20%)
Difficult defecation or constipation	5 (12.20%)
Prolapse of mass	15 (36.59%)
Tenesmus	7 (17.07%)
Abdominal pain	4 (9.76%)
Diarrhea	3 (7.32%)
No symptom	4 (9.76%)
Disease course, months (median, IQR)	12 (33)
Laboratory Results (n, %)	
Anemia	8 (19.51%)
Thrombocytosis	16 (39.02%)
Leukocytosis	1 (2.44%)
Elevated hs-CRP	3 (3/36, 8.33%)
Positive 13 C urea breath test	8 (8/13, 61.54%)
Positive serum antibody of *H. pylori*	0 (0/8, 0)
Imaging Results (n, %)	
Contrast-enhanced abdominal CT	
Benign lesions	10 (10/19, 52.63%)
Malignant lesions	5 (5/19, 26.32%)
Unclear	3 (3/19, 15.79%)
Normal	1 (1/19, 5.26%)
Contrast-enhanced abdominal MRI	
Benign lesions	19 (19/22, 86.36%)
Malignant lesions	3 (3/19, 13.64%)

IQR, interquartile range; hs-CRP, high-sensitivity C reactive protein; CT, computed tomography; MRI, magnetic resonance imaging.

Anemia: hemoglobin <120 g/L for male and <11 g/L for female; thrombocytosis: platelet >3*10^9/L; leukocytosis: white blood cell >10*10^9/L; elevated hs-CRP: hs-CRP > 3 mg/L.

^a^ some patients had more than one symptom.

### Endoscopic and pathological features

**FIGURE 1:**
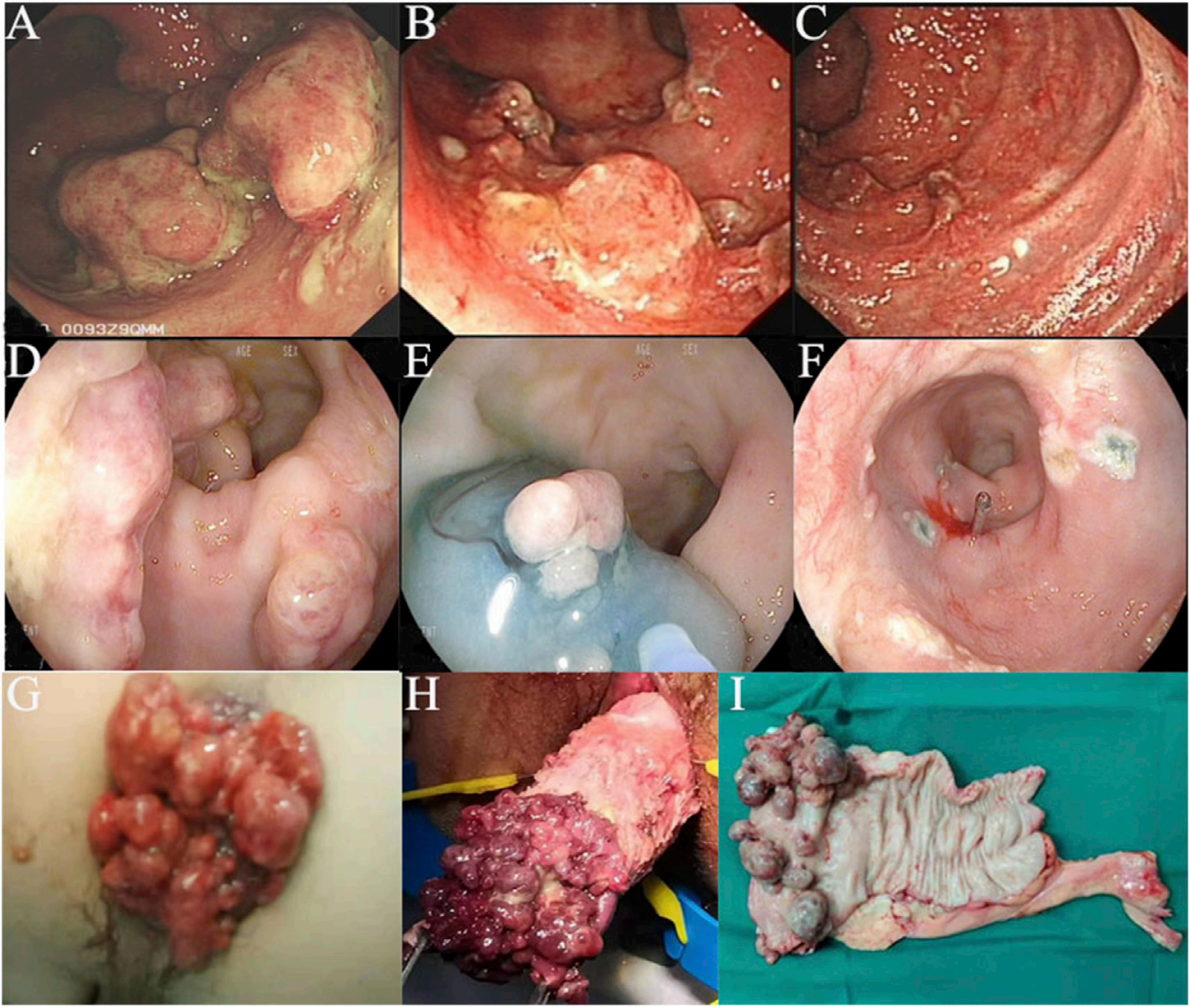
Colonoscopy images and surgical specimen of cap polyposis (CP): **(A–C)**, Patient No. 30, colonoscopy images before and after medical treatments; **(D–F)**, Patient No. 5, colonoscopy images showing endoscopic mucosal resection for endotherapy of CP; **(G–I)**, Patient No. 11, with prolapse of CP, which was treated by surgical resection.

**FIGURE 2:**
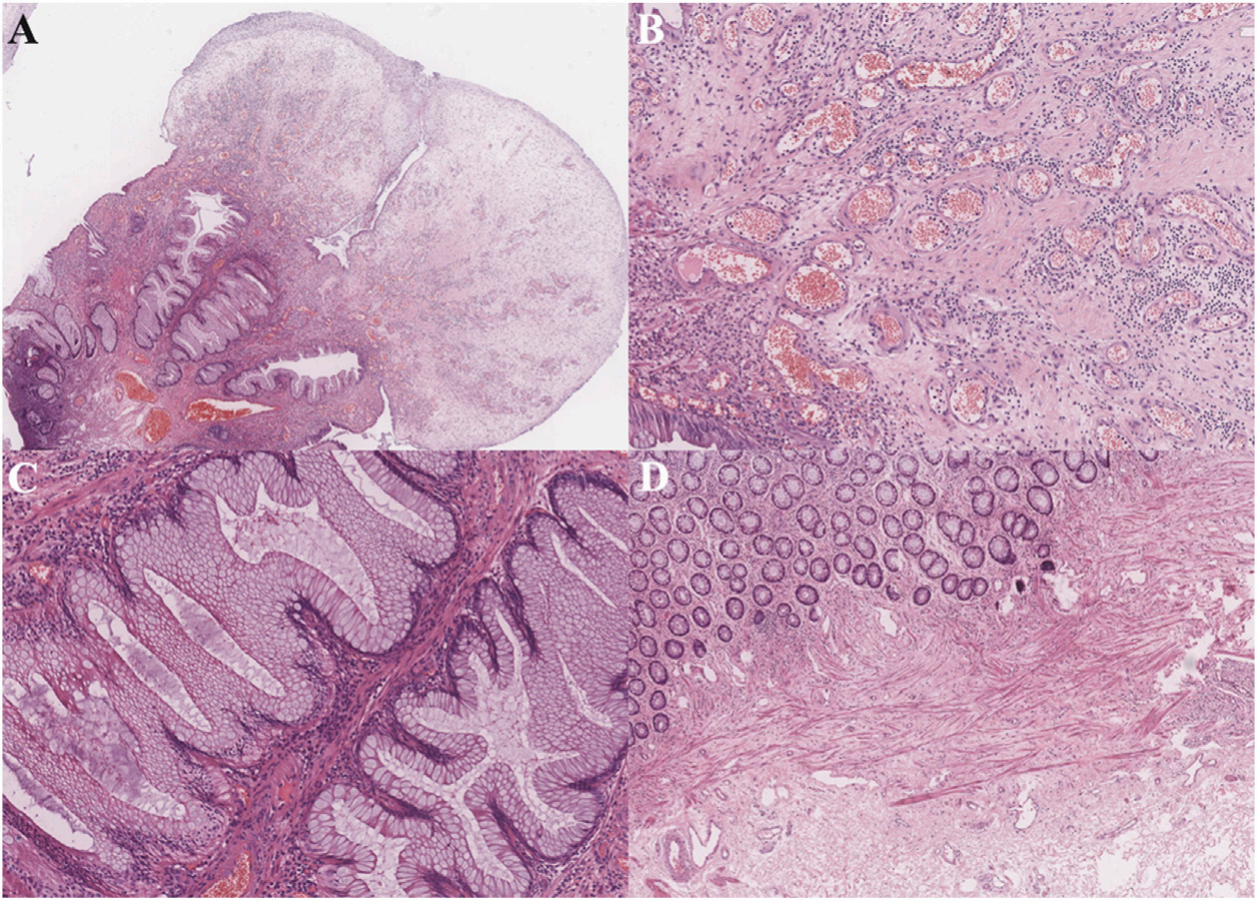
Pathological findings (hematoxylin and eosin staining.): **(A)** Prominent polypoid lesion seen covered by a cap of inflammatory granulation tissue (1X power); **(B)** Inflammatory cells immersed and inflammatory granulation tissue (10X power); **(C)** Goblet cell hyperplasia with serrated appearance, and cyst dilation with increased mucous secretion (10X power); **(D)** In other cases, ischemic changes could be seen with crypt atrophy and interstitial fibrous tissue hyperplasia (4X power).

According to the endoscopic findings, multiple erythematous, polyps covered by rich exudates of different sizes were frequently found (Figures 1A, B). For 22 patients, the involved site was the rectum alone, and for another 19 patients, both the anus and rectum were involved. In 11 patients, the number of polyps was less than 5, while for others, the number of polyps was ≥5. Pathology revealed polyps covered with fibrous purulent exudates and inflammatory granulation tissue forming a “cap” structure (Figures 2A, B), with crypt dilatation and mucous oversecretion within the crypts (Figure 2C); ischemic changes with crypt atrophy and interstitial fibrous tissue hyperplasia mimicking rectal prolapse could also be observed (Figure 2D).

### Treatments and outcomes

As no standard therapy has been recommended for CP, there were multiple and combined treatments for these patients. These treatments can be mainly divided into 3 categories: medical therapy (including observation, *H. pylori* eradication, corticoids enema, oral metronidazole, mesalazine anal plug/enema/oral, and biofeedback therapy), endotherapy [including polypectomy with snare and endoscopic mucosal resection (EMR)], and surgery (including transanal polypectomy, laparoscopic rectum resection, and laparoscopic rectal fixation). Further details are presented in Supplementary Table S1.

From the follow-up results, we found that medical therapy could help to diminish the size of the polyps but was difficult to resolve (Figure 1C); however, the patients’ symptoms could be resolved. Twelve patients received endotherapy (Figures 1D–F) and 23 patients received surgical resection (Figures 1G–I). We further compared their efficacy and safety. Both could resolve the polyps, but with high recurrence. Endotherapy and surgery were both safe, and the mortality rate was 0%. Only 1 patient (8.33%) who received endoscopic polypectomy with a snare had slight bleeding, and emergent colonoscopy revealed no signs of active bleeding; 1 patient (4.35%) who received transanal+ laparoscopic partial rectum resection had anastomotic leakage, and was treated by conservative methods; and another patient (4.35%) who received laparoscopic partial rectum resection+ ileostomy had anastomotic stricture, and was treated by endoscopic stricture dilation and incision.

**FIGURE 3:**
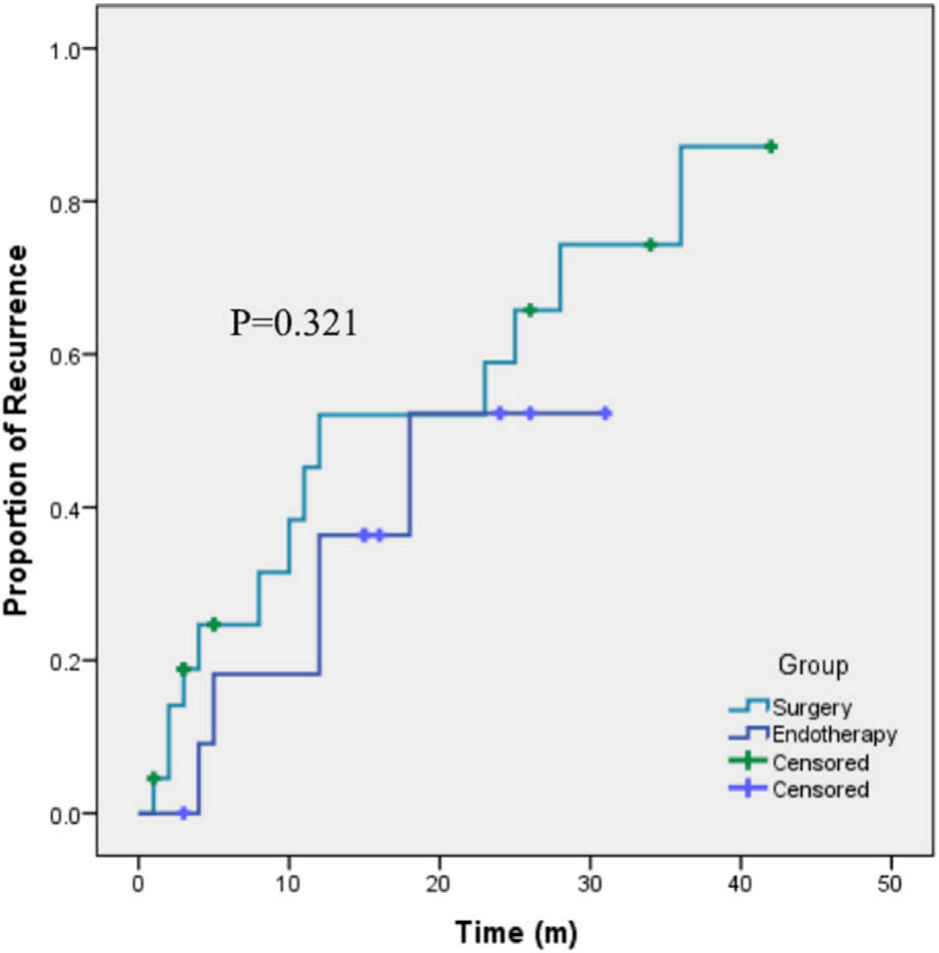
Kaplan-Meier curves showing the recurrence rates after endotherapy or surgery for the treatment of cap polyposis.

The patients who underwent polyp resection were followed for a median of 26 (IQR 19) months (1 patient who underwent surgical resection was lost). Eighteen patients (51.43%) experienced recurrence, and the recurrence rate was not significantly different between the two groups (41.67% vs. 59.09% *p*=0.331). Kaplan‒Meier curves (Figure 3) further supported that, the recurrence risk was not significantly different between the two kinds of therapy (*p*=0.321).

### Literature review

We also summarized the literature on this issue (Arana et al., 2014; Suzuki et al., 2014; Kim et al., 2017; Murata et al., 2017; Anuchapreeda et al., 2018; Batra et al., 2018; Okamoto et al., 2018; Tamura et al., 2018; Brunner et al., 2019; Monsalve et al., 2020), and evaluated the percentage of clinical improvement associated with these treatments (Table 2).

**TABLE 2:** Efficacy ratio by treatment in the previously published cases.

Treatments	Effective cases/total cases	Efficacy ratio (%)
Observation	6/7	85.7
Avoiding straining at defecation	2/5	40
Total parental nutrition	0/5	0
Metronidazole^a^	7/22	31.8
Quinolone	0/2	0
Steroids (oral)	2/17	11.8
Steroids (enema or suppo)	4/15	26.7
Aminosalicylates (oral)	0/24	0
Aminosalicylates (enema or suppo)	1/4	25
*Helicobacter pylori* eradication^b^	15/16	>93.8
Infliximab	2/5	40
Resection (endoscopic or surgical)	23/37	62.2

^a^ 1effective case used metronidazole+ amoxicillin.

^b^ 1 effective case combined steroids oral and enema, and 1 case failed was *H. pylori* negative.

## Discussion

CP is a relatively rare disease that is still not well recognized by physicians and surgeons, and currently, fewer than 100 cases have been reported in the literature (Aggarwal et al., 2021). In our study, most of the patients were very young, their median age was only 20 years, and some of them were significantly negatively affected by this disease, which impacted their normal work and study status. Hence, we believe that more attention should be devoted to this disease. Here, we share our experience regarding CP in our hospital to contribute additional data to the existing literature.

The majority of the patients were male, which was in accordance with previous literature (Papaconstantinou et al., 2012; Li et al., 2013; Suzuki et al., 2014). In 2012, Papaconstantinou et al. described the cases of CP (Papaconstantinou et al., 2012), and most of the patients were older than 50 years of age; however, Brunner et al. reported that children or younger people may also develop CP (Brunner et al., 2019). The laboratory, and imaging characteristics of CP are not specific. A portion of them had anemia and thrombocytosis, while leukocytosis or elevated hs-CRP was not common. Additionally, we should be aware that a certain number of patients are misdiagnosed with malignant lesions on contrast-enhanced CT or MRI, with rates of 26.32% and 13.64%, respectively. Rectal cancer usually presents as a mass and seldomly multiple polyps (unless familial adenomatous polyposis), and repeated biopsy does not support malignant changes. If differentiation is still difficult, then a large piece of the polyp should be resected to help make a diagnosis.

IBD is also an important differential diagnosis, especially in children: it was the initial diagnosis in 75% of the reports on children (Kreisel et al., 2017). However, endoscopically, ulcerative colitis has a background mucous change, while CP is usually characterized by multiple polyps covered by fibrinopurulent exudates with normal mucosa between the polyps, and they have different pathologic features.

The relationship between CP and mucosal prolapse syndrome (MPS) is still debated. Some patients with CP have symptoms of prolapse. Cambell et al. suggested that abnormal colonic motility may lead to prolapse of the mucosa at the apices of transverse mucosal folds and cause ischemic changes (Campbell et al., 1993). The pathology included fibromuscular obliteration of the lamina propria, granulation tissue, and elongated, hyperplastic glands. These features can also appear in MPS. However, they have some differences. First, fibromuscular obliteration is more marked in cap polyposis; second, CP can be found in both the colon and rectum, while MPS is usually confined to the rectum; third, the two diseases have different endoscopic ultrasound sonography images, as CP shows significant thickening of the mucosa, whereas MPS has remarkable thickening of the submucous (Hizawa et al., 1994; Shimizu et al., 2002; Li et al., 2013).

To date, there is no standard or optimal therapy for CP, as its cause remains unclear. According to the previously published literature, various kinds of treatments, including medical therapies (such as observation, steroids, aminosalicylates, infliximab, metronidazole, and *H. pylori* eradication), and endoscopic and surgical resection have been attempted. However, the clinical outcomes were heterogeneous. Some patients experienced spontaneous remission, while some needed surgical resection and still experienced recurrence (Suzuki et al., 2014). Some investigators believe that, in adults, polypectomy should be performed to alleviate symptoms. However, in children, medical treatments are preferred. If the disease persists or recurs with medical treatment, then we should consider resection (Ng et al., 2004; Brunner et al., 2019). In our study, we further compared the long-term effectiveness of endotherapy and surgical resection, although with a small number of patients, we found that the recurrence risk was not significantly different. However, we combined several different means of endotherapy or surgical resection in the analysis, and we think that if we could accumulate more patients, then we may find a certain means of resection with the best short-term and long-term outcomes.

Based on the literature review (Arana et al., 2014; Suzuki et al., 2014; Bordeianou et al., 2017; Kim et al., 2017; Murata et al., 2017; Anuchapreeda et al., 2018; Batra et al., 2018; Okamoto et al., 2018; Tamura et al., 2018; Brunner et al., 2019; Monsalve et al., 2020) and our own data, we hypothesized that, if the patient was *H. pylori* positive, then *H. pylori* eradication combined with other therapies might be useful. There were 7 patients who were tested to have *H. pylori* infection; 5 of them improved after *H. pylori* eradication, the other two were lost to follow-up. For patients who was negative in *H. pylori* test, although various means tried, the outcome was not as good. Moreover, for those with rectal prolapse, if rectal prolapse is not treated, CP seems to recur repeatedly.

The goal of CP treatment is another factor that must be considered. During follow-up, some patients had no symptoms, but colonoscopy still revealed small polyps (or recurrence of polyps). These patients felt very well and did not want to see the doctors for further treatment at that time. Should patients receive treatments until the disappearance of all the polyps, until the disappearance of all the symptoms or until the symptoms do not influence the patient’s work and life? The above questions remain to be answered. The natural course of CP is largely unknown, and if asymptomatic or slightly symptomatic CP is left untreated, the chance and risk of developing malignant lesions are also unknown. In this study, no malignant lesions were observed during follow-up. In the future, by obtaining more information on the abovementioned aspects, we can determine the goal of CP treatment and the follow-up strategy.

There are several limitations in this study. First, due to the rarity of CP, it was very difficult to conduct a prospective, large sample-size study, so we only retrospectively reviewed the clinical data of these patients, and because of the retrospective nature of the study, the treatment options were not chosen following a preestablished algorithm, and the pretreatment conditions were difficult to evaluate in different groups. Second, as no standard therapy for CP is recommended, the treatments used vary greatly among patients, and even within the same patients during different periods; moreover, combined treatments are sometimes used. It was very difficult to simply generalize these therapies into several kinds and evaluate their efficacy. Third, as not all patients underwent colonoscopy during follow-up, in this study, recurrence was defined as polyp recurrence on endoscopy or digital rectal examination, or the reappearance of symptoms. However, some patients might have no symptoms, but colonoscopy can reveal polyp recurrence. If we defined recurrence as polyp recurrence on endoscopy, then the recurrence rates in this study were underestimated. (Bordeianou et al., 2017).

In conclusion, CP is a disease with an increasing incidence rate that affects mostly young males and it easily recurs. Endoscopic and imaging features can mimic IBD or rectal cancer, and their relationship with MPS is still under debate. The clinical improvement resulting from medical treatments was not satisfactory and was inconsistent. Endotherapy or surgical resection can remove the polyposis and provide temporary relief, but the recurrence rates are high, with no difference between the two methods.

## Data Availability

The raw data supporting the conclusion of this article will be made available by the authors, without undue reservation.
