# Supplementary material for: Clinical, endoscopic, pathological characteristics and management of cap polyposis: experience from a Tertiary Hospital in China
Source: Front Pharmacol. 2024 May 9;15:1391367. doi: 10.3389/fphar.2024.1391367 (PMC11111883; doi:10.3389/fphar.2024.1391367)
Supplement: Supplementary file 1 [file Table1.pdf]

Table S1 Treatments and Subsequent Outcomes of the Patients

Pt	Sex	Age	Treatment	Status and subsequent treatments
1	M	11	Surgery	Recurrence after 25 m→ medical therapy
2	M	20	Endotherapy	Recurrence after 4 m→ endotherapy→ recurrence after 20 m→ medical therapy
3	F	46	Endotherapy	No recurrence for 3 m
4	M	18	Surgery + medical therapy	Recurrence after 11 m→ surgery→ recurrence after 25 m→ surgery + medical therapy
5	M	18	Surgery in other hospital	Recurrence after 8 m→ surgery→ recurrence after 2 m→ surgery→ recurrence after 4 m→ surgery→ recurrence after 11 m→ medical therapy→ endotherapy→ recurrence after 15 m→ endotherapy + medical therapy
6	M	18	Surgery	No recurrence for 3 m
7	M	24	Surgery in other hospital	Recurrence after 2 m→ endotherapy+ medical therapy→ mild hematochezia after 1 m
8	M	16	Surgery	Polyps still existed
9	M	18	Surgery	No recurrence for 5 m
10	F	15	Medical therapy	Lost
11	M	15	Medical therapy→ polyps get smaller→ laparoscopic +surgery	Recurrence after 10 m
12	M	33	Medical therapy	Got smaller and symptoms disappeared→ mucous stool and hematochezia after 10 m
13	M	15	Surgery in other hospital	Recurrence after 36 m→ biofeedback therapy→ no symptoms for 10 m
14	M	28	Surgery in other hospital	Recurrence after 23 m→ surgery → no recurrence for 14 m
15	M	31	Endotherapy	No symptom for 15 m
16	F	33	Endotherapy	No symptom for 15 m
17	M	15	Medical therapy→ polyps get smaller→ endotherapy	Recurrence after 12 m→ medical therapy→ no recurrence for 14 m
18	M	22	Surgery + medical therapy	No recurrence for 5 m
19	F	32	Endotherapy	No recurrence for 16 m
20	M	19	Surgery	Recurrence after 12 m→ no symptom, observation
21	M	20	Endotherapy + medical therapy	Recurrence after 5 m→ no symptom, observation

22	M	31	Medical therapy	Polyps still existed
23	M	19	Surgery	No recurrence for 34 m
24	M	20	Surgery + medical therapy	Recurrence after 4 m→ surgery→ recurrence after 20 m→ medical therapy
25	M	6	Endotherapy	No symptom for 24 m
26	M	2	Endotherapy	Hematochezia after 18 m→ observation
27	M	23	Medical therapy→ without getting smaller→ surgery	Recurrence after 28 m→ surgery
28	M	21	Endotherapy	No recurrence for 26 m
29	M	19	Surgery + medical therapy	Recurrence after 2 m→ no symptom, observation
30	M	29	Medical therapy	Polyps got smaller and no symptom for 15 m
31	M	14	Surgery in other hospital	Recurrence after 3 m→ surgery→ recurrence after 9 m→ surgery→ medical therapy→ no symptom for 15 m
32	M	11	Endotherapy	No symptom for 31 m
33	M	24	Surgery	No recurrence for 26 m
34	M	30	Surgery	No symptom for 42 m
35	M	31	Surgery + medical therapy	Lost
36	M	12	Surgery in other hospital	Recurrence after 1 m→ surgery
37	M	16	Endotherapy in other hospital	Recurrence after 12 m→ surgery→ recurrence after 47 m
38	M	33	Surgery	No symptom for 1 m
39	M	16	Surgery	No symptom for 3 m
40	F	32	Surgery	No symptom for 3 m
41	M	26	Observation	Lost

HP, *Helicobacter pylori*
